# Photothermal Conversion Efficiency of Silver and Gold Incorporated Nanosized Apatites for Biomedical Applications

**DOI:** 10.1021/acsomega.3c04809

**Published:** 2023-10-27

**Authors:** Nataliia D. Pinchuk, Agnieszka Paściak, Grzegorz Paściak, Paulina Sobierajska, Jacek Chmielowiec, Oleksii Bezkrovnyi, Piotr Kraszkiewicz, Rafal J. Wiglusz

**Affiliations:** † Institute of Low Temperature and Structure Research, Polish Academy of Sciences, Wroclaw 50-422, Poland; ‡ Frantsevich Institute for Problems of Materials Science of NAS of Ukraine, Kyiv 03142, Ukraine; § Wroclaw University of Science and Technology, The Faculty of Fundamental Problems of Technology, 50-370 Wroclaw, Poland; ⊥ Department of Organic Chemistry, Bioorganic Chemistry and Biotechnology, Silesian University of Technology, Krzywoustego 4, 44-100 Gliwice, Poland

## Abstract

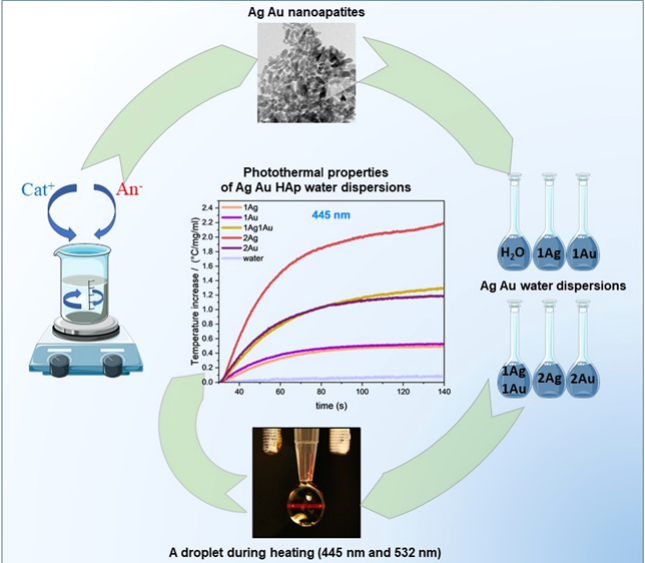

The aim of this research was to investigate the photothermal ability of nanocrystalline hydroxyapatite (nHAp) incorporated with silver and gold. It was studied by using a recently developed technique evaluating the photothermal conversion efficiency. The heating performance of aqueous dispersions was examined under 445 and 532 nm excitation. The largest increase in temperature was found for the 2% Ag-nHAp and reached above 2 °C per mg/mL of sample (445 nm) under 90 mW laser continuous irradiation and an external light-to-heat conversion efficiency of 0.11 L/g cm. The obtained results have shown a new functionality of nanosized apatites that has not been considered before. The studied materials have also been characterized by XRPD, TEM, BET, and UV–Vis techniques. Finally, in this work, a new idea for their application was proposed: photothermal therapy.

## 1 Introduction

An important issue of biomedical materials is the development of various bioactive properties that can perform several functions simultaneously. Particular attention has been paid to multifunctional materials for both therapy and diagnostics (theranostics). Among them, nanosized hydroxyapatite (nHAp) shows promising features with potential use in implantology. Hydroxyapatite is of continuing interest to researchers because it occurs naturally in the bone structure and can be a component of implants.^1^ There are different methods for the synthesis of hydroxyapatite (hydrothermal, precipitation, sol–gel, biomimetic, and their combination).^2^ The use of various technological parameters provides opportunities to obtain nanosized apatites (nAp) that show different properties.^3−7^ Research on materials based on nHAp focuses primarily on the possibility of their use as a basis for replacing damaged human bone tissue after injuries or various diseases. However, in medical practice, the use of materials that not only replace bone tissue but also have other bioactive properties, including drug delivery, as well as the diagnosis of diseases are desired. Recently, cofunctional materials for both therapy and diagnostics have been extensively studied.^7^ The various combinations of bioactive features that will simultaneously perform several functions are very up to date. However, inflammatory, infectious, and oncological processes also occur in bone tissue. In this case, it is very important that the material for replacing the bone not only restores its defective areas but also has anti-inflammatory, antimicrobial, antitumor, and other properties that prevent pathological processes in the human body. In this context, various dopants are used to obtain specific properties of the final material.

Over the last few years, hydroxyapatites doped as well as codoped with silver and gold ions have been developed.^7−19^ They have improved antibacterial properties and can be used for theranostics. Moreover, the doping of hydroxyapatite with noble metals can open a new possibility for its application in diseases that are treated with photothermal therapy. Noble metal nanoparticles have unique physical, chemical, and biological properties. For example, gold and silver nanoparticles (AuNPs and AgNPs) have increased chemical, biological, and antimicrobial activity against various types of pathogens (bacteria and viruses) of various origins. The effect of nanosized noble particles is superior to the effect of large-sized silver particles. Also, the properties of noble metal nanoparticles depend significantly on their shape. Nanoparticles in the form of nanospheres, nanorods, nanocages, nanosized shells, and others have different absorption properties.^20^ The use of nanostructured materials composed of noble metal NPs and nHAp takes full advantage of their unique features. Our last report^7^ indicated that the nanosized apatites in combination with noble metals (Ag, Au, Pd) strongly inhibited adhesion and biofilm production by the selected drug-resistant strains of *Enterococcus faecalis* and *Staphylococcus aureus*. At the same time, the tested materials did not show a cytotoxic effect on fibroblasts during incubation with bacterial biofilms. The application of noble metal nanomaterials can resolve the microbial resistance issue due to its unique features such as smaller size than bacteria, high specific surface area (strong surface interaction), surface plasmon resonance, and stability.^21^

A feature of noble metals with different morphologies (e.g., spheres or rods) is the presence of an absorption band in the visible or NIR range of the electromagnetic radiation spectrum. The high efficiency of excitation of plasmon waves on the surface of gold/silver nanoparticles allows them to be used in photothermal therapy. When gold or silver nanoparticles are exposed to light from a specific resonant wavelength, strong absorption or scattering occurs. The degree of absorption depends primarily on the morphology and dielectric environment of the gold nanoparticles. It is well-known that nanoparticles of gold and silver can generate heat due to light absorption.^22,23^ The heating mechanism in noble metal nanoparticles is based on the surface plasmon resonance.^24^ As a result of this phenomenon, electromagnetic radiation induces the oscillations of conductive free electrons, resulting in heat generation. The unique optical properties of the plasmonic structures have induced interest in using them for different biomedical applications.

Nanosized heaters (NHs) have shown potential in the treatment of cancer through photothermal therapy (PTT). PTT takes advantage of the fact that cancer cells are more vulnerable than healthy cells to overheating beyond 41 °C (hyperthermia range) or can be destroyed by thermal ablation (temperatures above 48 °C).^23^ The authors of ref (22) reported that the light-to-heat energy transfer efficiency of gold nanoparticles (AuNPs) increases as the particle size decreases. Reference (25) demonstrates that polydispersity has a significant impact on the optical performance of plasmonic nanostructures. In ref (26) photothermal ablative therapy of Au-PEG clusters on animals was applied, resulting in successful intertumoral treatments. Yang et al.^27^ prepared doxorubicin-loaded alendronate-modified hollow gold nanoparticles for bone-targeted chemo-photothermal therapy. The as-prepared silver nanocages (AgNCs) with high photothermal conversion efficiency exhibited excellent photothermal stability and could induce effective thermal ablation of tumors under NIR laser irradiation.^28^ The PTT of HAp-Au is a method of promoting wound healing by using a photothermal therapy-assisted nanosized catalytic antibacterial system utilizing a polydopamine coating on hydroxyapatite incorporated with gold nanoparticles.^11^ This method is safe, rapid, and effective against bacteria and stimulates tissue-repairing-related gene expression to facilitate the formation of granulation tissues and collagen synthesis. In another study,^29^ NIR-responsive gelatin/HAp/GNR composite microspheres were prepared and found to have a strong photothermal property that could be controlled through adjustment of NIR irradiation time and GNR content. In ref (30) a bifunctional gelatin/methacrylated chondroitin sulfate hydrogel hybrid gold nanorod and nanohydroxyapatite (nHAp) were constructed to eradicate residual tumors after surgery and bone regeneration. This GNRs/HAp hybrid hydrogel displayed regulated high temperatures in response to different power densities of NIR laser irradiation.

Nanomaterials with different chemical compositions and characteristics demonstrate different photothermal capabilities, which should be evaluated in a way that allows them to be compared between different laboratories.^23,31^ Recently, a technique for measuring the photothermal properties of colloidal dispersions of materials was developed, which makes it possible to determine in an accurate way the efficiency of light-to-heat conversion, both internal (iHCE), indicating what percentage of the absorbed energy will be converted to heat, and external (eHCE), describing the heat-generating capacity of the light-absorbing material.^23,31^ The methodology employed was originally used to evaluate the photothermal properties of materials including plasmonic, semiconductor, iron oxide, and lanthanide-doped nanomaterials but can also be applied to any other type of nanoparticles.^23,31^ Investigation of agglomerated gold nanoparticle clusters embedded in polyelectrolyte films demonstrates that the type of medium must be considered when describing light-mediated heating of gold nanoparticle clusters, which are fixed in a matrix surrounded by medium.^32^ To minimize the impact of the medium on the results, Paściak et al. recommended a comparison of colloidal materials dispersed in an aqueous medium.^23,31^

The main aim of our research was to investigate photothermal properties of nanocrystalline apatites incorporated with silver (Ag^+/0^) and gold (Au^+/0^) materials mainly used and prevously tested by us as bone implants.^7^ This approach makes it possible to realize new properties arising from the mutual influence of the components to offer new original ideas for the treatment of diseases of the human skeletal system, including cancer. We examined how the dopant of gold and silver affects the temperature rise and internal and external light-to-heat conversion efficiency. Measurements were carried out using a previously proposed technique for light-to-heat conversion efficiency determination^31^ related to the use of aqueous dispersions. To obtain the stable aqueous dispersions of the hydroxyapatite, we proposed multidirectional action, chemical surface modification using a sodium citrate agent together with ultrasound support.

## 2 Materials and Methods

### 2.1 Synthesis of Au–Ag Incorporated Nanosized Apatites

The protocol for the synthesis of the materials presented in this paper was described in our previous article.^7^ Briefly, the Ag^+/^Ag^0^ or/and Au^+/^Au^0^ nanosized apatites were synthesized by the coprecipitation method. The concentration of silver and gold was set at the level of 1–2 mol % to the overall molar content of Ca^2+^ cations: 1 mol % Ag^+^; 1 mol % Au^+^; 2 mol % Ag^+^; 2 mol % Au^+^; 1 mol % Ag^+^; and 1 mol % Au^+^. The water-soluble HAuCl_4_ was obtained using a mixture of nitric acid and hydrochloric acid (1:3). Nanosized apatites with gold also had some substitution of OH^–^ ions for Cl^–^ related to the synthesis method as well as silver (Ag^0^) and gold (Au^0^) precipitates.

The substrates (Ca(NO_3_)_2_·4H_2_O, 99% Acros Organics, Schwerte, Germany; (NH_4_)_2_HPO_4_, ≥98% Avantor Performance Materials Poland S.A, Gliwice, Poland; AgNO_3_, ≥99.9% Avantor Performance Materials Poland S.A, Gliwice, Poland; Au, Mennica-Metale, Radzymin, Poland) were dissolved, forming a homogeneous mixture. To achieve a pH of 10, an ammonia solution was added to the mixture, which then was heated to 70 °C for 3 h. The precipitated product was dried at 90 °C for 24 h. Finally, a heat treatment was conducted at 450 °C for 3 h.

### 2.2 Preparation of Colloidal Dispersions for Photothermal Measurements

**Figure 1:**
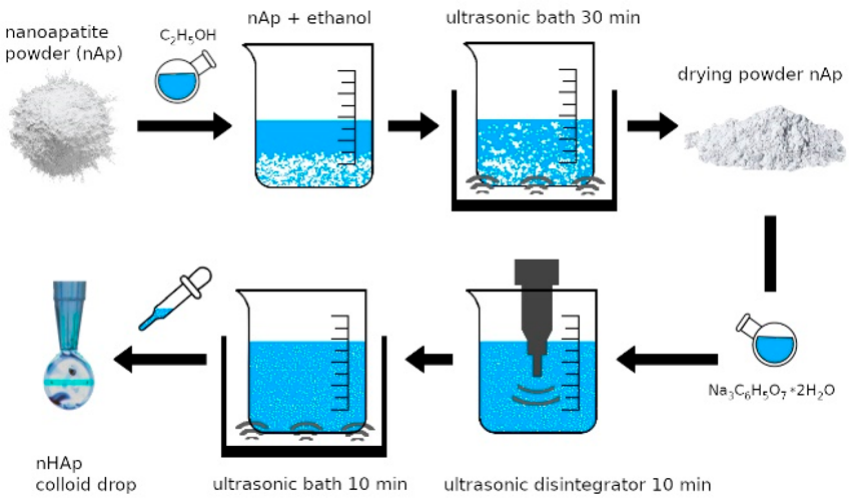
Schematic presentation of the technological process of nanosized apatite colloidal dispersions.

The colloidal suspension was necessary to compare the photothermal properties of the test samples with those of other photothermal agents. However, obtaining the stable dispersion of hydroxyapatite is a rather complicated process, which involves the use of mechanical action (stirring, milling), dispersants,^33,34^ or ultrasonic shredding, as well as their combinations. Moreover, the role of heat treatment of hydroxyapatite powder prior to suspension preparation.^35^ It is also crucial to use the relevant dispersion stabilizer. Such a stabilizing agent might be sodium citrate, which is well-known as a biocompatible dispersant.^36−38^ Colloidal dispersions were prepared based on silver/gold incorporated nanosized apatite powders. To obtain stable colloidal dispersions of nanoapatite, trisodium citrate dihydrate C_6_H_5_Na_3_O_7_·2H_2_O (TSC) was used as the dispersion agent. Nanoapatite powders were pretreated with ethanol in an ultrasonic bath (30 min) and dried at room temperature. After that 0.1 g of the powders was mixed with 10 mL of trisodium citrate solution (0.01 wt %) and additionally sonicated in an ultrasonic disperser UZDN M900-T (22 kHz, 900 W, Akademprylad, Ukraine) with the following parameters: power 60%, 3 s “working”, 1 s “pause”. The dispersions were left undisturbed for 1 day. Samples for photothermal measurements were then taken from the top of the dispersions. Before photothermal measurements, the obtained colloidal dispersions were placed in an ultrasonic bath (10 min). The preparation of nanosized apatite aqueous colloidal dispersions for photothermal measurements is presented in Figure 1.

### 2.3 Characterization of Materials

Initial nanopowders and colloidal dispersions were characterized. A PANalytical X’Pert Pro X-ray diffractometer (Malvern Panalytical Ltd., Malvern, UK) with Cu–K radiation at the 2θ range from 10° to 60° (exposure time of 2 h) was applied to determine the structure and crystallinity. The obtained diffraction patterns were juxtaposed with those from the Inorganic Crystal Structure Database (ICSD).

The element concentrations were determined using a scanning electron microscope (SEM, FEI Nova NanoSEM 230, Hillsboro, OR, USA) with an energy-dispersive X-ray spectrometer (EDS, Genesis XM4, Austin, TX, USA).

The UV–Vis spectra were recorded on an Agilent Cary 5000 UV–Vis–NIR spectrophotometer (Agilent Technologies, Santa Clara, CA, USA) with a spectral bandwidth of 1 nm in the range of 200–800 nm (50,000–12,500 cm^–1^).

The morphology of the samples was determined by transmission electron microscopy (TEM), using a Philips CM-20 SuperTwin instrument operating at 160 kV. Specimens were prepared by dispersing the sample in methanol and putting a droplet of the suspension on a copper microscope grid covered with carbon. Samples were then dried and purified in oxygen/hydrogen plasma in a plasma cleaner.

Nitrogen adsorption–desorption isotherms were determined at 77 K on a Micromeritics ASAP 2020 instrument. Before measurements, the samples were degassed under vacuum at 200 °C for 4 h. The specific surface area (SBET) was calculated using the Brunauer–Emmett–Teller (BET) method (*p*/*p*_0_ ranged from 0.05 to 0.2). The total pore volume (*V*_p_) was calculated from the amount of nitrogen adsorbed at a *p*/*p*_0_ of 0.995.

### 2.4 Photothermal Conversion Efficiency Evaluation

To determine the photothermal properties of the nanoapatites, the methodology described in detail in the work of Paściak et al.^23,31^ was used. Briefly, when illuminating the sample with laser radiation, a temperature curve is obtained, which can be fitted using the Wang model:^39^
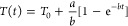
1The internal light-to-heat efficiency (iHCE) can be determined from the equation:
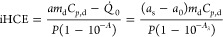
2where *a* is the parameter a from a temperature curve determined independently for sample (s) and water solvent (0); *m*_d_ is the mass of a droplet sample; *C*_*p*,d_ is the heat capacity of water; *P* is laser power; and *A*_λ_ is absorbance determined in situ.

The external efficiency (eHCE) was determined from the equation:
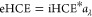
3where *a*_λ_ (L/g cm) is a mass absorption coefficient determined from the Lambert–Beer Law.

The iHCE and eHCE were evaluated in a system in which the sample was in the form of a drop,^23,31^ and its temperature was monitored by a thermal imaging camera (FLIR T540) during laser irradiation. Optical power during irradiation was measured with two power meters, the one placed behind the droplet and the one used as a reference, to determine the actual power (S120C head photodiodes and PM100USB power meter, Thorlabs). Droplet volumes were estimated based on the thermal imaging camera photos and were 12 ± 1 μL (droplet diameter ∼2.6 mm). The 445 and 532 nm laser diodes (1.5 and 3 W, Changchun New Industries Optoelectronics Technology Co., Ltd.) were used as an irradiation source, and the irradiation power on the sample was 90 mW. The samples were diluted so as not to exceed a temperature increment of 5 °C, to reduce droplet evaporation.

## 3 Results and Discussions

### 3.1 Physicochemical Properties of Obtained Materials

#### 3.1.1 XRPD Analysis

**Figure 2:**
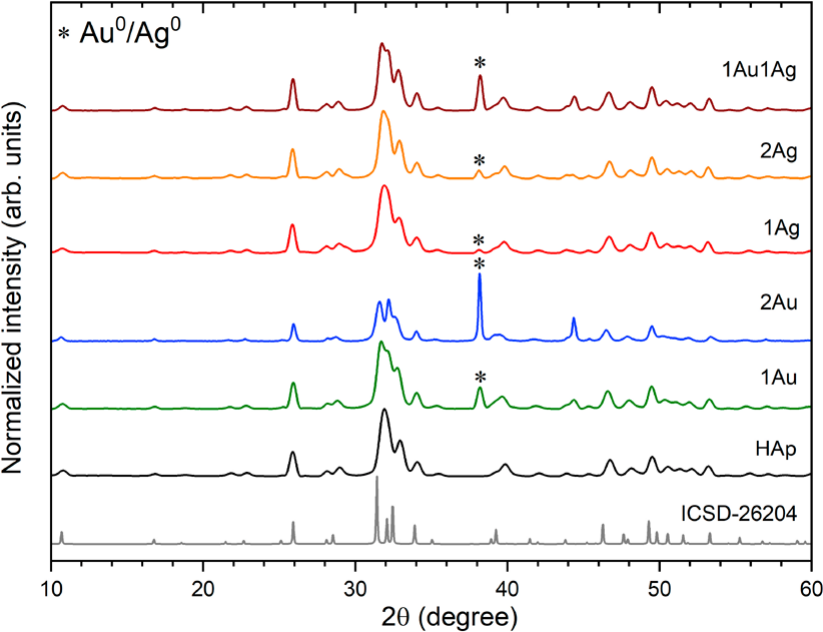
XRPD diffraction patterns of the nanoapatites, pure and incorporated with silver and gold after incubation in aqueous dispersion of sodium citrate and ultrasonic treatment.

To determine the crystallinity and phase structure, the nanoapatites incorporated with 1% Ag^+/^Ag^0^, 1% Au^+/^Au^0^, 2% Ag^+/^Ag^0^, 2% Au^+/^Au^0^, and 1% Ag^+/^Au^0^–1% Au^+/^Au^0^ were characterized by the X-ray powder diffraction technique (XRPD). The diffraction patterns in Figure 2 show wide well-developed peaks confirming the formation of nanosized crystalline materials which is in line with the previous research.^7^

Despite the samples being suspended in a sodium citrate solution and treated by ultrasound (ultrasonic disperser) there are no differences in diffraction patterns compared to those of the initial materials.^7^ The treated materials retain the crystallographic phase of apatite and still contain an additional phase of Ag^0^ (ICSD-22434^40^ /Au^0^ (ICSD-52249^41^), which is visible as an extra peak at 2θ of about 38°. Moreover, in the case of materials with Au^+^/Au^0^, the mixed apatite phase (OH–Cl–Ap) is present because of the use of a gold chloride precursor for the synthesis (confirmed in our previous work by SEM-EDS measurements).^7^

#### 3.1.2 SEM-EDS Analysis

**Figure 3:**
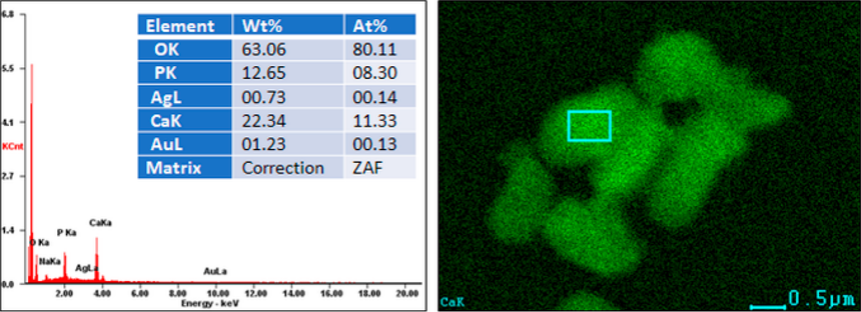
EDS spectra of the nHAp codoped with silver and gold after incubation in aqueous dispersion of sodium citrate and ultrasonic treatment.

The contents of chemical elements in the obtained materials after sodium citrate and ultrasonic treatment were analyzed by the SEM-EDS method (Figure 3). Here, we present the representative results for the nHAp codoped with silver and gold. The calculated average percentage of silver and gold was 1.21 mol % and 1.12 mol %, respectively, which is consistent with the theoretical values and with the results obtained for the material before dispersion in sodium citrate.

#### 3.1.3 UV–Vis Analysis

**Figure 4:**
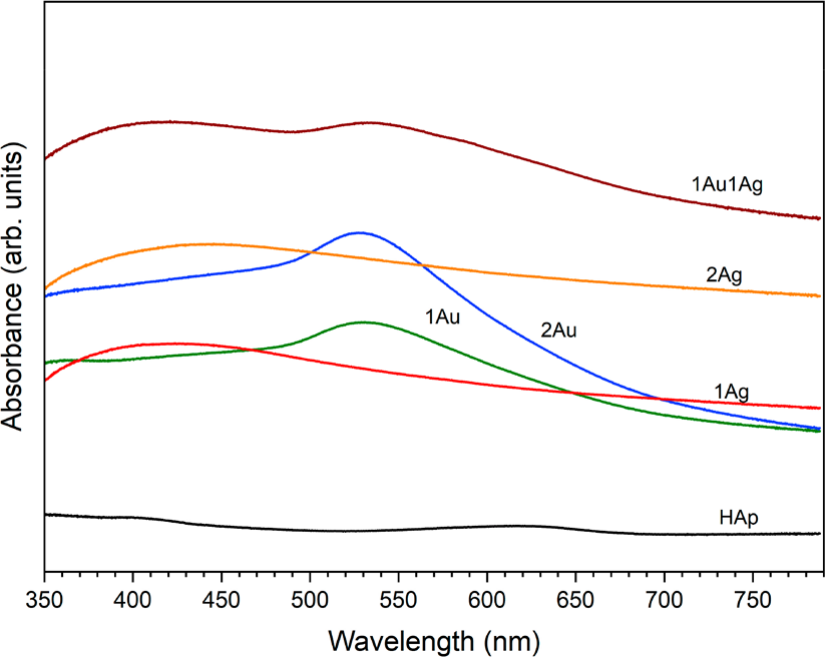
UV–Vis spectra of the nanoapatites, pure and incorporated with silver and gold, after incubation in an aqueous dispersion of sodium citrate and ultrasonic treatment.

UV–Vis spectra of the pure and Ag/Au-incorporated nanosized apatites after incubation in aqueous dispersion of sodium citrate and ultrasound treatment are shown in Figure 4. The samples were dried before measurements. The resulting spectra are like initial spectra of the same powders described in the previous publication.^7^ From this, it can be concluded that the incubation in aqueous dispersions treated with sodium citrate as well as treated by ultrasound had no effect on the absorption properties of nanosized apatite powders.

The spectra show surface plasmon resonance peaks characteristic of metal nanoparticles. The spectra of nanosized apatites with the addition of gold are characterized by peaks around 530–540 nm that indicate the presence of Au nanoparticles.^42^ The spectra of the samples with silver, in comparison with pure apatite, are characterized by a wide band with a maximum at about 420–430 nm, which confirms the presence of silver nanoparticles.^43^ For the spectrum of the sample containing 1% silver and 1% gold, both peaks can be seen, which are characteristic of the presence of both gold and silver nanoparticles. When samples are excited within the absorption band ranges, heat generation can be expected to occur. Sharp displacement visible at 350 nm is related to the detector change.

#### 3.1.4 TEM Analysis

**Figure 5:**
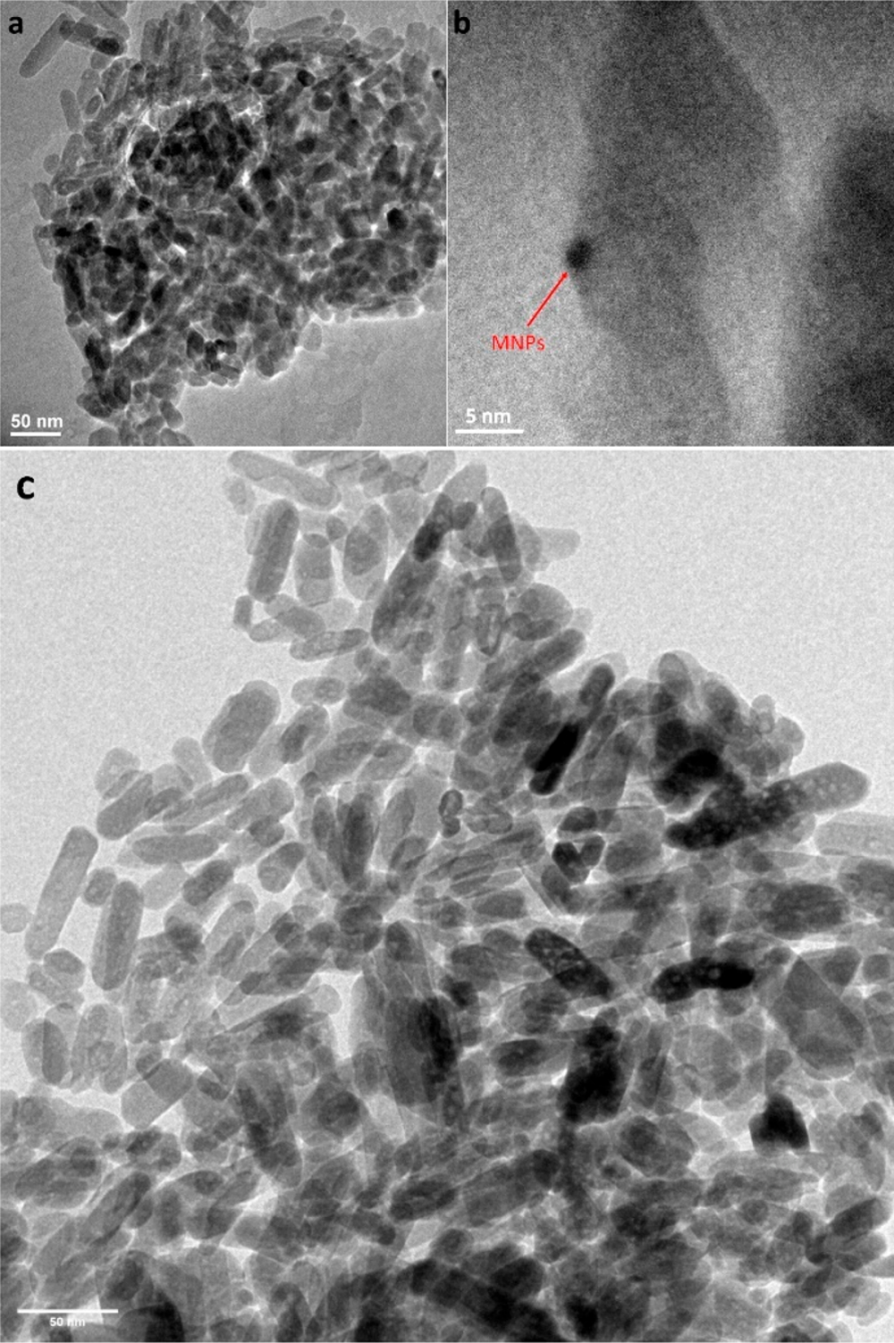
TEM image of the nanoapatite particles incorporated with 1% silver and 1% gold (450 °C) after colloidal dispersion (a). HRTEM image reveals the decoration of apatite particles with metal nanoparticles (MNPs) (b). TEM image of the initial Ag–Au–nHAp nanoparticles (450 °C) (c).

**Figure 6:**
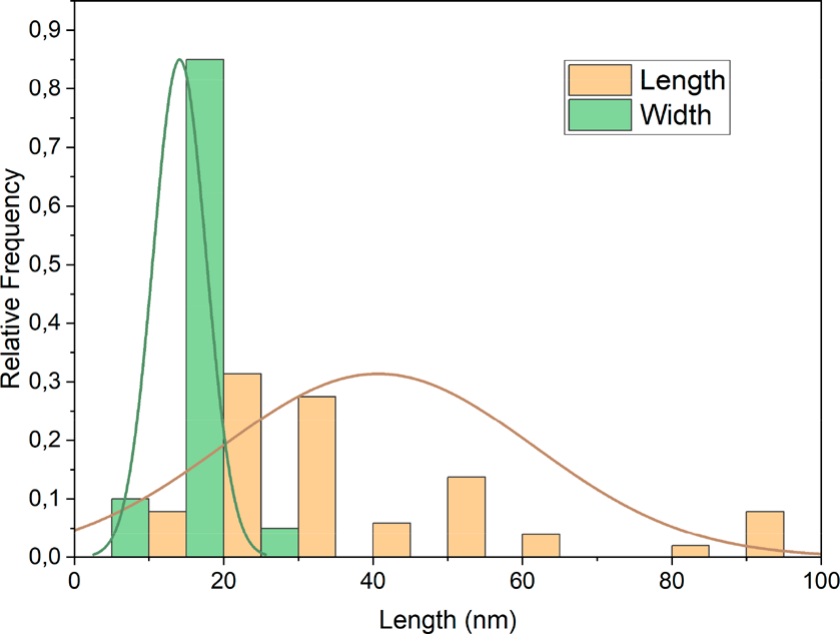
Grain size distribution of the nanosized apatites incorporated with 1% silver and 1% gold (450 °C).

Also, the size of the nanoparticles is important both for the delivery of drugs and for the occurrence of surface plasmon resonance. As can be seen in TEM images, Ag–Au–nHAp particles (450 °C) have a regular elongated round shape with dimensions from 10 to 92 nm in length and from 9 to 25 nm in width (Figures 5 and 6). The average particle size, in this case, is 42 nm for length and 15 nm for width (Figure 6). High-resolution TEM measurements show apatite particles with a metal nanoparticle a few nm in size (Figure 5b) as previously described by other authors.^44,45^ In comparison to the literature, it should be noted that in our study the concentrations of gold and silver ions are significantly lower (several orders of magnitude). Metallic particles were formed during the synthesis of Ag^+^- and Au^+^-doped nanosized nHAp. Consequently, a portion of the ions was incorporated into the nHAp structure (Ca^2+^ ions were substituted by Ag^+^ and Au^+^ ions), while the remaining ions constitute metallic precipitates, particularly Au^0^ (manifested as a surface plasmon resonance). Notably, the previous work^7^ includes SEM images of these materials for reference. The rationale for our choice of minimum dopant concentration (1–2 mol % per Ca^2+^ ions) is based on our main goals: achievement of efficient light-to-heat conversion while ensuring the material remains noncytotoxic. Moreover, it was demonstrated^7^ that nanosized apatites combined with noble metals (Ag^0^, Au^0^, Pd^0^) effectively impede adhesion and biofilm formation in drug-resistant *Enterococcus faecalis* and *Staphylococcus aureus* strains. Furthermore, these materials exhibited no cytotoxic effects on fibroblasts when exposed to bacterial biofilms.

TEM images of Ag–Au–nHAp (450 °C) after incubation in sodium citrate solution and treated by ultrasound, washed, centrifuged, and dried at 60 °C showed that the sizes of nanoparticles are the same size as the initial nanomaterial (Figure 5a,c). Thus, the use of the above-described method for obtaining a colloidal dispersion of nanosized apatite, despite the use of a sufficiently high content of sodium citrate, makes it possible to preserve the nanostructure of the powder. Wiglusz et al. concluded that 100–200 nm sized particles may pass through the pores of vessels, but an ideal drug carrier needs to possess the loading matrices, surface modifications, and targeting functionalization.^46^ Thus, the sizes of the nanoparticles in the investigated materials for drug delivery systems are optimal.

The initial powder is visually indistinguishable from the powder that has been stored in solution with sodium citrate for a long time, subjected to strong ultrasound, as well as centrifuged and dried at a temperature of 60 °C (∼24 h) (Figure 5).

#### 3.1.5 BET Analysis

The calculated average surface area and pore volume based on the adsorption–desorption isotherms of nanosized apatite samples are shown in Table 1. The introduction of gold or silver into hydroxyapatite generally leads to a material with a smaller specific surface area compared to pure hydroxyapatite. The largest calculated specific surface area was specified for pure hydroxyapatite nanoparticles (64.1 m^2^/g). For a sample with the addition of silver, nHAp: 2% Ag+/Ag^0^, the specific surface area is reduced by about 20% compared with pure hydroxyapatite. For the sample with the addition of 2% Au+/Au^0^ into the apatite, the specific surface area is reduced by about 30% compared to that of pure hydroxyapatite.

**Table 1:** Average Surface Area and Pore Volume of Obtained Nanoapatites

Material	Average surface area *S*_BET_, m^2^/g	Pore volume *V*_p,_ cm^3^/g
HAp (450 °C)	64.1	0.345
HAp 2%Ag (450 °C)	52.3	0.243
HAp 2% Au (450 °C)	44.7	0.332

The introduction of gold or silver into hydroxyapatite generally also leads to a material with a smaller pore volume compared with pure hydroxyapatite. As in the case of specific surface area, pure hydroxyapatite has the largest pore volume (0.345 m^2^/g). However, in the case of materials with noble metals, a different dependence is observed. For gold (nHAp: 2% Au^+^/Au^0^), the pore volume is 96% of the value for pure hydroxyapatite. In the case of silver (nHAp: 2% Ag+/Ag^0^), the pore volume decreases significantly and equals 70% compared with the pure hydroxyapatite. It is expected that this reduction of specific surface area and porosity is due to the loading of silver and gold in nanocrystals into the pores of the hydroxyapatite.

### 3.2 Photothermal Properties of Incorporated Nanosized Apatites

**Figure 7:**
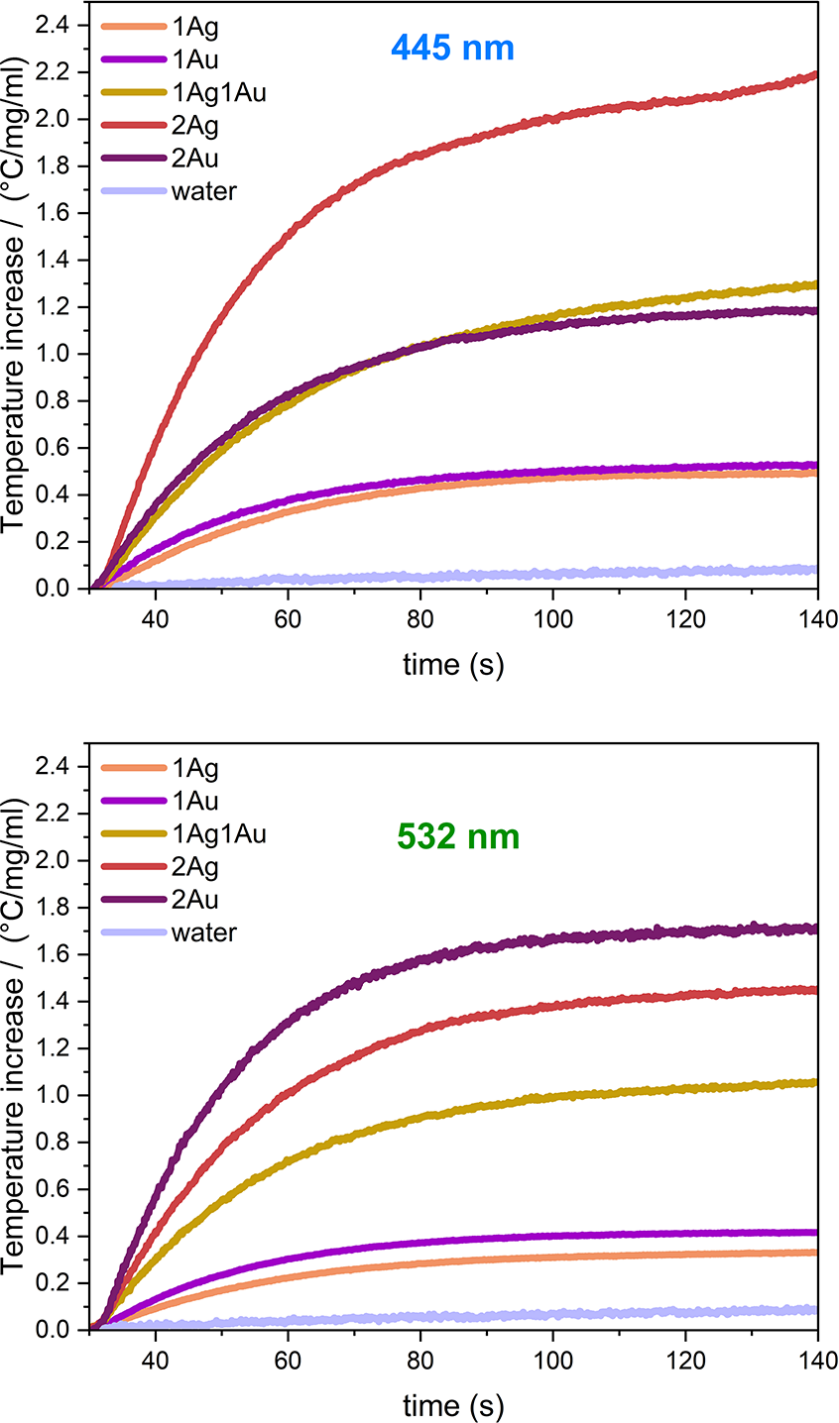
Temperature increase normalized per 1 mg/mL of sample as a function of time of nanoapatite aqueous dispersions and water under 445 and 532 nm irradiation.

The photothermal properties of the obtained nanosized apatite colloidal dispersions with 1–2% of noble metals (1% Ag^+^/Ag^0^; 1% Au^+^/Au^0^; 2% Ag^+^/Ag^0^; 2% Au^+^/Au^0^; 1% Ag^+^/Ag^0^–1% Au^+^/Au^0^) were investigated under 445 and 532 nm irradiation. Since the temperature rise is strongly concentration-dependent, the temperature curves are presented after normalization for 1 mg/mL nanomaterial concentration (Figure 7). Obtaining stable colloids is confirmed by stable optical power behind the droplet during the illumination.

All samples supported with silver and gold exhibit significant photothermal properties. The temperature increment is greater for samples with higher concentrations of noble metals. At 445 nm, better results are obtained for materials with silver, while at 532 nm, better results are obtained for materials with gold. This effect can be explained by the absorption properties of these materials: the absorption maximum for gold occurs at around 530 nm, while for silver, it occurs around 430 nm. We did not observe a significant temperature increase for sodium citrate alone or for pure nanohydroxyapatite (see Table 2).

**Table 2:** Results of Photothermal Measurements of Investigated Samples^a^

Colloidal dispesions	*c* (mg/mL)	d*T* 445 nm	iHCE 445 nm (%)	eHCE (L/g cm) 445 nm	*c* (mg/mL)	d*T* 532 nm	iHCE 532 nm (%)	eHCE (L/g cm) 532 nm
nHAp	3.8	0.3	0.4	–	–	–	–	–
TSC	1.3	0.1	–	–	–	–	–	–
nAp 1 mol % Ag^+^	4.3	2.2	5.9	0.027	8.5	2.8	8.0	0.022
nAp 1 mol % Au^+^	3.9	2.1	6.3	0.031	9.2	3.9	12.2	0.033
nAp 2 mol % Ag^+^	1.8	4.1	18.8	0.110	1.8	2.7	18.9	0.065
nAp 2 mol % Au^+^	1.8	2.2	11.6	0.055	1.8	3.1	14.8	0.089
nAp 1 mol % Au^+^, 1 mol % Ag^+^	1.9	2.4	11.0	0.046	1.9	2.0	16.4	0.043

a *c*, concentration; *d*T, temperature rise after stabilization; iHCE, internal light-to-heat conversion efficiency; eHCE, external light-to-heat conversion efficiency.

Hence, we consider that the measured temperature increments come mostly from the absorption of gold and/or silver. A cosupported sample shows better photothermal properties than samples supported with only one type of noble metal. However, when the total concentration of silver/gold is considered, it turns out that the coincorporated sample is comparable to or worse than such materials. However, it is found that silver and gold, even at such a low concentration, can induce a significant temperature rise. Calculations indicate a small (<20%) internal HCE. This is due to the significant scattering properties of the samples. This scattering is believed to occur due to the presence of hydroxyapatite, which exhibits scattering properties in an aqueous environment. For example, the optical power behind the water droplet at 445 nm excitation was 72.6 mW, behind the HA sample with 1% Ag^+^/Ag^0^and 22.3 mW, and 36.7 mW, behind the pure HA (at similar concentration). Interestingly, similar iHCEs were obtained for both wavelengths.

Typically, iHCEs were higher for samples with a higher concentration of noble metals. However, the eHCE parameter is of much greater application significance as it considers the absorption coefficient of the material. At 532 nm, the highest eHCE was reached by a sample doped with 2% gold, which is consistent with the absorption spectrum of gold at approximately 530 nm (Figure 3). However, the highest eHCE (0.11 L/g·cm) was obtained for the sample with 2% Ag^+^/Ag^0^ at 445 nm. This value is comparable to those obtained for gold nanorods (0.18 L/g cm) and carbon dots (0.34 L/g cm) at 445 nm.^23^

## 4 Conclusions

Colloidal dispersions suitable for measuring the photothermal properties of Ag or Au (1–2%)-incorporated apatite nanoparticles were successfully prepared using sodium citrate and ultrasonic treatment. An innovative and reliable measurement system^23^ has been used to observe light to heat conversion for the obtained materials. The light-to-heat conversion efficiency measurements of these colloidal dispersions have been performed with two laser diodes: 445 and 532 nm. The temperature increase and internal and external light-to-heat conversion efficiencies (iHCE and eHCE) of the obtained dispersions were determined. The largest increase in temperature was found for the sample with 2% silver and reached 2.2 °C per mg/mL of sample. Similar results were obtained when determining iHCE of the samples: the maximum iHCE (∼19%) and eHCE (0.11L/gcm) were found for the 2% Ag - nanoapatite. The eHCE parameter that we determined allows a quantitative comparison of our nanomaterials with materials used as nanoheaters and proves that hydroxyapatite in combination with small amounts of gold or silver is promising for photothermal therapy.

## ABBREVIATIONS

nHAp: nanohydroxyapatite
iHCE: internal light-to-heat efficiency
eHCE: external light-to-heat efficiency
PTT: photothermal therapy
NPs: nanoparticles
TEM: transmission electron microscopy
SEM: scanning electron microscopy
EDS: energy dispersive spectroscopy
XRPD: X-ray powder diffraction

## References

[ref1] Le Geros R. Z. Properties of Osteoconductive Biomaterials: Calcium Phosphates. Clin Orthop Relat Res. 2002, 395, 81–98. 10.1097/00003086-200202000-00009. 11937868

[ref2] Mohd Pu’ad N. A. S.; Abdul Haq R. H.; Mohd Noh H.; Abdullah H. Z.; Idris M. I.; Lee T. C. Synthesis Method of Hydroxyapatite: A Review. Mater. Today Proc. 2020, 29, 233–239. 10.1016/j.matpr.2020.05.536.

[ref3] Barbosa A. A.; Júnior S. A.; Mendes R. L.; de Lima R. S.; de Vasconcelos Ferraz A. Multifunctional Hydroxyapatite with Potential for Application in Theranostic Nanomedicine. Materials Science and Engineering: C 2020, 116, 111227 10.1016/j.msec.2020.111227. 32806238

[ref4] Huang S.-M.; Liu S.-M.; Ko C.-L.; Chen W.-C. Advances of Hydroxyapatite Hybrid Organic Composite Used as Drug or Protein Carriers for Biomedical Applications: A Review. Polymers (Basel) 2022, 14 (5), 976 10.3390/polym14050976. 35267796 PMC8912323

[ref5] George S. M.; Nayak C.; Singh I.; Balani K. Multifunctional Hydroxyapatite Composites for Orthopedic Applications: A Review. ACS Biomater Sci. Eng. 2022, 8 (8), 3162–3186. 10.1021/acsbiomaterials.2c00140. 35838237

[ref6] Bonany M.; Pérez-Berná A. J.; Dučić T.; Pereiro E.; Martin-Gómez H.; Mas-Moruno C.; van Rijt S.; Zhao Z.; Espanol M.; Ginebra M.-P. Hydroxyapatite Nanoparticles-Cell Interaction: New Approaches to Disclose the Fate of Membrane-Bound and Internalised Nanoparticles. Biomaterials Advances 2022, 142, 213148 10.1016/j.bioadv.2022.213148. 36274359

[ref7] Paluch E.; Sobierajska P.; Okińczyc P.; Widelski J.; Duda-Madej A.; Krzyżanowska B.; Krzyżek P.; Ogórek R.; Szperlik J.; Chmielowiec J.; Gościniak G.; Wiglusz R. J. Nanoapatites Doped and Co-Doped with Noble Metal Ions as Modern Antibiofilm Materials for Biomedical Applications against Drug-Resistant Clinical Strains of Enterococcus Faecalis VRE and Staphylococcus Aureus MRSA. Int. J. Mol. Sci. 2022, 23 (3), 1533 10.3390/ijms23031533. 35163457 PMC8836119

[ref8] Mocanu A.; Furtos G.; Rapuntean S.; Horovitz O.; Flore C.; Garbo C.; Danisteanu A.; Rapuntean G.; Prejmerean C.; Tomoaia-Cotisel M. Synthesis; Characterization and Antimicrobial Effects of Composites Based on Multi-Substituted Hydroxyapatite and Silver Nanoparticles. Appl. Surf. Sci. 2014, 298, 225–235. 10.1016/j.apsusc.2014.01.166.

[ref9] Silva-Holguín P. N.; Reyes-López S. Y. Synthesis of Hydroxyapatite-Ag Composite as Antimicrobial Agent. Dose-Response 2020, 18 (3), 155932582095134 10.1177/1559325820951342. PMC7485164 32952484

[ref10] Bolli E.; Kaciulis S.; Mezzi A.; Ambrogi V.; Nocchetti M.; Latterini L.; Di Michele A.; Padeletti G. Hydroxyapatite Functionalized Calcium Carbonate Composites with Ag Nanoparticles: An Integrated Characterization Study. Nanomaterials 2021, 11, 2263 10.3390/nano11092263. 34578579 PMC8469523

[ref11] Xu X.; Liu X.; Tan L.; Cui Z.; Yang X.; Zhu S.; Li Z.; Yuan X.; Zheng Y.; Yeung K. W. K.; Chu P. K.; Wu S. Controlled-Temperature Photothermal and Oxidative Bacteria Killing and Acceleration of Wound Healing by Polydopamine-Assisted Au-Hydroxyapatite Nanorods. Acta Biomater 2018, 77, 352–364. 10.1016/j.actbio.2018.07.030. 30030176

[ref12] Ahmed M. K.; Ramadan R.; Afifi M.; Menazea A. A. Au-Doped Carbonated Hydroxyapatite Sputtered on Alumina Scaffolds via Pulsed Laser Deposition for Biomedical Applications. Journal of Materials Research and Technology 2020, 9 (4), 8854–8866. 10.1016/j.jmrt.2020.06.006.

[ref13] Fatimah I.; Citradewi P. W.; Yahya A.; Nugroho B. H.; Hidayat H.; Purwiandono G.; Sagadevan S.; Mohd Ghazali S. A. I. S.; Ibrahim S. Biosynthesized Gold Nanoparticles-Doped Hydroxyapatite as Antibacterial and Antioxidant Nanocomposite. Mater. Res. Express 2021, 8 (11), 115003 10.1088/2053-1591/ac3309.

[ref14] Dolai J.; Biswas A.; Ray R.; Jana N. R. Enhanced Piezocatalysis by Calcium Phosphate Nanowires via Gold Nanoparticle Conjugation. ACS Appl. Mater. Interfaces 2022, 14 (23), 26443–26454. 10.1021/acsami.2c05036. 35666829

[ref15] Radhakrishnan R.; Kannan K.; Kumaravel S.; Thiripuranthagan S. Oxidative Esterification of Furfural over Au-Pd/HAP-T and Au-Ag/HAP-T Bimetallic Catalysts Supported on Mesoporous Hydroxyapatite Nanorods. RSC Adv. 2016, 6 (51), 45907–45922. 10.1039/C6RA07614A.

[ref16] Kim H.; Mondal S.; Jang B.; Manivasagan P.; Moorthy M. S.; Oh J. Biomimetic Synthesis of Metal-Hydroxyapatite (Au-HAp, Ag-HAp, Au-Ag-HAp): Structural Analysis, Spectroscopic Characterization and Biomedical Application. Ceram. Int. 2018, 44 (16), 20490–20500. 10.1016/j.ceramint.2018.08.045.

[ref17] Kumar V. B.; Khajuria D. K.; Karasik D.; Gedanken A. Silver and Gold Doped Hydroxyapatite Nanocomposites for Enhanced Bone Regeneration. Biomedical Materials 2019, 14 (5), 055002 10.1088/1748-605X/ab28e4. 31185462

[ref18] Vukomanović M.; Repnik U.; Zavašnik-Bergant T.; Kostanjšek R.; Škapin S. D.; Suvorov D. Is Nano-Silver Safe within Bioactive Hydroxyapatite Composites?. ACS Biomater Sci. Eng. 2015, 1 (10), 935–946. 10.1021/acsbiomaterials.5b00170. 33429525

[ref19] Chen K.; Ustriyana P.; Moore F.; Sahai N. Biological Response of and Blood Plasma Protein Adsorption on Silver-Doped Hydroxyapatite. ACS Biomater Sci. Eng. 2019, 5 (2), 561–571. 10.1021/acsbiomaterials.8b00996. 33405820

[ref20] Jauffred L.; Samadi A.; Klingberg H.; Bendix P. M.; Oddershede L. B. Plasmonic Heating of Nanostructures. Chem. Rev. 2019, 119 (13), 8087–8130. 10.1021/acs.chemrev.8b00738. 31125213

[ref21] Ye L.; Cao Z.; Liu X.; Cui Z.; Li Z.; Liang Y.; Zhu S.; Wu S. Noble Metal-Based Nanomaterials as Antibacterial Agents. J. Alloys Compd. 2022, 904, 164091 10.1016/j.jallcom.2022.164091.

[ref22] Jiang K.; Smith D. A.; Pinchuk A. Size-Dependent Photothermal Conversion Efficiencies of Plasmonically Heated Gold Nanoparticles. J. Phys. Chem. C 2013, 117 (51), 27073–27080. 10.1021/jp409067h.

[ref23] Paściak A.; Marin R.; Abiven L.; Pilch-Wróbel A.; Misiak M.; Xu W.; Prorok K.; Bezkrovnyi O.; Marciniak Ł.; Chanéac C.; Gazeau F.; Bazzi R.; Roux S.; Viana B.; Lehto V.-P.; Jaque D.; Bednarkiewicz A. Quantitative Comparison of the Light-to-Heat Conversion Efficiency in Nanomaterials Suitable for Photothermal Therapy. ACS Appl. Mater. Interfaces 2022, 14 (29), 33555–33566. 10.1021/acsami.2c08013. 35848997 PMC9335407

[ref24] Amendola V.; Pilot R.; Frasconi M.; Maragò O. M.; Iatì M. A. Surface Plasmon Resonance in Gold Nanoparticles: A Review. J. Phys.: Condens. Matter 2017, 29 (20), 203002 10.1088/1361-648X/aa60f3. 28426435

[ref25] Qin Z.; Wang Y.; Randrianalisoa J.; Raeesi V.; Chan W. C. W.; Lipiński W.; Bischof J. C. Quantitative Comparison of Photothermal Heat Generation between Gold Nanospheres and Nanorods. Sci. Rep 2016, 6 (1), 29836 10.1038/srep29836. 27445172 PMC4956767

[ref26] Schwartz-Duval A. S.; Konopka C. J.; Moitra P.; Daza E. A.; Srivastava I.; Johnson E. v.; Kampert T. L.; Fayn S.; Haran A.; Dobrucki L. W.; Pan D. Intratumoral Generation of Photothermal Gold Nanoparticles through a Vectorized Biomineralization of Ionic Gold. Nat. Commun. 2020, 10.1038/s41467-020-17595-6. PMC7483505 32913195

[ref27] Yang L.; Hu Y.; Liu Y.; Liu Y.; Miao S.; Li Z.; Xu B.; Shen Y. Preparation and *in Vitro* Evaluation of Doxorubicin Loaded Alendronate Modified Hollow Gold Nanoparticles for Bone-Targeted Chemo-Photothermal Therapy. Materials Express 2020, 10 (11), 1950–1959. 10.1166/mex.2020.1862.

[ref28] Bian K.; Zhang X.; Liu K.; Yin T.; Liu H.; Niu K.; Cao W.; Gao D. Peptide-Directed Hierarchical Mineralized Silver Nanocages for Anti-Tumor Photothermal Therapy. ACS Sustain Chem. Eng. 2018, 6 (6), 7574–7588. 10.1021/acssuschemeng.8b00415.

[ref29] Chen S.; Du X.; Wang T.; Jia L.; Huang D.; Chen W. Synthesis of Near-Infrared Responsive Gold Nanorod-Doped Gelatin/Hydroxyapatite Composite Microspheres with Controlled Photo-Thermal Property. Ceram. Int. 2018, 44 (1), 900–904. 10.1016/j.ceramint.2017.10.020.

[ref30] Liao J.; Shi K.; Jia Y.; Wu Y.; Qian Z. Gold Nanorods and Nanohydroxyapatite Hybrid Hydrogel for Preventing Bone Tumor Recurrence via Postoperative Photothermal Therapy and Bone Regeneration Promotion. Bioact Mater. 2021, 6 (8), 2221–2230. 10.1016/j.bioactmat.2021.01.006. 33553811 PMC7829101

[ref31] Paściak A.; Pilch-Wróbel A.; Marciniak Ł.; Schuck P. J.; Bednarkiewicz A. Standardization of Methodology of Light-to-Heat Conversion Efficiency Determination for Colloidal Nanoheaters. ACS Appl. Mater. Interfaces 2021, 13 (37), 44556–44567. 10.1021/acsami.1c12409. 34498862 PMC8461604

[ref32] Hühn D.; Govorov A.; Gil P. R.; Parak W. J. Photostimulated Au Nanoheaters in Polymer and Biological Media: Characterization of Mechanical Destruction and Boiling. Adv. Funct Mater. 2012, 22 (2), 294–303. 10.1002/adfm.201101134.

[ref33] Rodríguez-Lorenzo L. M.; Vallet-Regí M.; Ferreira J. M. F. Colloidal Processing of Hydroxyapatite. Biomaterials 2001, 22 (13), 1847–1852. 10.1016/S0142-9612(00)00366-5. 11396889

[ref34] Huang Y.; Yan S.; Zhang S.; Yin Q.; Chen X.; Wu W. D. Spray Dried Hydroxyapatite-Based Supraparticles with Uniform and Controllable Size and Morphology. Colloids Surf. B Biointerfaces 2022, 217, 112610 10.1016/j.colsurfb.2022.112610. 35700565

[ref35] Amin A. M. M.; Besisa D. H. A.; El-Amir A. A. M.; Zaki Z. I.; Ahmed Y. M. Z. Role of Heat Treatment of Hydroxyapatite Powder Prior to Suspension Preparation on the Suspension Flow Behavior. Open Ceramics 2022, 9, 100239 10.1016/j.oceram.2022.100239.

[ref36] Li C.; Zhao L.; Han J.; Wang R.; Xiong C.; Xie X. Synthesis of Citrate-Stabilized Hydrocolloids of Hydroxyapatite through a Novel Two-Stage Method: A Possible Aggregates-Breakdown Mechanism of Colloid Formation. J. Colloid Interface Sci. 2011, 360 (2), 341–349. 10.1016/j.jcis.2011.04.059. 21565359

[ref37] Yang H.; Hao L.; Du C.; Wang Y. A Systematic Examination of the Morphology of Hydroxyapatite in the Presence of Citrate. RSC Adv. 2013, 3 (45), 23184 10.1039/c3ra44839h.

[ref38] Jin X.; Zhuang J.; Zhang Z.; Guo H.; Tan J. Hydrothermal Synthesis of Hydroxyapatite Nanorods in the Presence of Sodium Citrate and Its Aqueous Colloidal Stability Evaluation in Neutral PH. J. Colloid Interface Sci. 2015, 443, 125–130. 10.1016/j.jcis.2014.12.010. 25544318

[ref39] Wang X.; Li G.; Ding Y.; Sun S. Understanding the Photothermal Effect of Gold Nanostars and Nanorods for Biomedical Applications. RSC Adv. 2014, 4 (57), 30375–30383. 10.1039/C4RA02978J.

[ref40] Swathi S.; Arun K.; Dzubinska A.; Reiffers M.; Nagalakshmi R. Systematic Investigations on the Magnetic Properties of Moderate Heavy Fermion CeAg0.68Si1.32 Alloy. Physica B Condens Matter 2019, 575, 411679 10.1016/j.physb.2019.411679.

[ref41] Owen E.A.; Yates E.L. Precision Measurements of Crystal Parameters. London, Edinburgh, and Dublin Philosophical Magazine and Journal of Science 1933, 15 (98), 472–488. 10.1080/14786443309462199.

[ref42] An X.; Kays J. C.; Lightcap I. v.; Ouyang T.; Dennis A. M.; Reinhard B. M. Wavelength-Dependent Bifunctional Plasmonic Photocatalysis in Au/Chalcopyrite Hybrid Nanostructures. ACS Nano 2022, 16 (4), 6813–6824. 10.1021/acsnano.2c01706. 35349253 PMC9676104

[ref43] Gevorgyan S.; Schubert R.; Falke S.; Lorenzen K.; Trchounian K.; Betzel C. Structural Characterization and Antibacterial Activity of Silver Nanoparticles Synthesized Using a Low-Molecular-Weight Royal Jelly Extract. Sci. Rep 2022, 12 (1), 14077 10.1038/s41598-022-17929-y. 35982108 PMC9388513

[ref44] Bee S.-L.; Bustami Y.; Ul-Hamid A.; Lim K.; Abdul Hamid Z. A. Synthesis of Silver Nanoparticle-Decorated Hydroxyapatite Nanocomposite with Combined Bioactivity and Antibacterial Properties. J. Mater. Sci. Mater. Med. 2021, 32 (9), 106 10.1007/s10856-021-06590-y. 34426879 PMC8382650

[ref45] Ni Z.; Gu X.; He Y.; Wang Z.; Zou X.; Zhao Y.; Sun L. Synthesis of Silver Nanoparticle-Decorated Hydroxyapatite (HA@Ag) Poriferous Nanocomposites and the Study of Their Antibacterial Activities. RSC Adv. 2018, 8 (73), 41722–41730. 10.1039/C8RA08148D. 35558815 PMC9091964

[ref46] Liu T.-M.; Conde J.; Lipiński T.; Bednarkiewicz A.; Huang C.-C. Revisiting the Classification of NIR-Absorbing/Emitting Nanomaterials for in Vivo Bioapplications. NPG Asia Mater. 2016, 8 (8), 295 10.1038/am.2016.106.

